# Molecular Pathogenesis of Inherited Platelet Dysfunction

**DOI:** 10.3390/biom15111528

**Published:** 2025-10-30

**Authors:** Agustín Rodríguez-Alén, Antonio Moscardó, José M. Bastida, José Rivera

**Affiliations:** 1 Servicio de Hematología, Hospital Universitario de Toledo, 45007 Toledo, Spain; arodrigueza@sescam.jccm.es; 2 Servicio de Hematología, Hospital Universitario y Politécnico La Fe, Instituto de Investigación Sanitaria La Fe, 46001 Valencia, Spain; moscardo_ant@gva.es; 3 Servicio de Hematología, Complejo Asistencial Universitario de Salamanca (CAUSA), Instituto de Investigación Biomédica de Salamanca (IBSAL), Universidad de Salamanca (USAL), 37008 Salamanca, Spain; chema@usal.es; 4 Servicio de Hematología, Hospital Universitario Morales Meseguer, Centro Regional de Hemodonación, Universidad de Murcia, IMIB-Pascual Parrilla, CIBERER-ISCIII, 30003 Murcia, Spain

**Keywords:** inherited platelet function disorders, Glanzmann thrombasthenia, congenital platelet signaling defects, platelet granule deficiency, Hermansky–Pudlak syndrome

## Abstract

Inherited platelet function disorders (IPFD) are characterized by normal platelet count and morphology but impaired function due to pathogenic variants in genes encoding membrane receptors, granule constituents, or intracellular signaling proteins. Glanzmann’s thrombasthenia, the most representative IPFD, results from *ITGA2B* or *ITGB3* mutations that disrupt the αIIbβ3 integrin complex, producing severe mucocutaneous bleeding. Advances in molecular genetics have expanded the IPFDs landscape to include defects in other platelet receptors (Glycoprotein (GP)-VI, P2Y_12_, and thromboxane A_2_[TxA_2_]-R), signaling mediators (RASGRP2, FERMT3, G-protein regulators, PLC, and TxA_2_ pathway enzymes), and granule biogenesis disorders such as Hermansky–Pudlak and Chediak–Higashi syndromes. High-throughput sequencing technologies, including long-read approaches, have greatly improved diagnostic yield and clarified genotype–phenotype correlations. Clinically, bleeding severity varies from mild to life-threatening, and management relies on antifibrinolytics, desmopressin, or platelet transfusion; recombinant activated factor VII and hematopoietic stem cell transplantation are reserved for selected cases. Emerging strategies such as gene therapy and bispecific antibodies that link platelets and coagulation factors represent promising advances toward targeted and preventive treatment. A better knowledge of the clinical features and understanding molecular pathogenesis of IPFDs not only enhances diagnostic precision and therapeutic options but also provides key insights into platelet biology, intracellular signaling, and the broader mechanisms of human hemostasis.

## 1. Preamble

In this review we will focus on the description of the main inherited platelet function disorders (IPFD) without alteration, in general, of the number or morphology of platelets. The best known of these, and perhaps the most serious, is Glanzmann’s thrombasthenia, which is why we dedicate special attention to it. In addition, there are other important pathologies related to alterations at the level of various platelet receptors, signal transmission systems, or the content and release of granules [1,2,3]. The clinical, laboratory and molecular characterization of patients with these diseases is helping to improve our knowledge of platelet function and discover new therapeutic targets.

These IPFD are therefore a group of rare pathologies, although the exact prevalence of many of them is unknown. Overall, it is estimated that they may affect up to 1 in 10,000 people, as common as von Willebrand disease (VWD). It should be noted that patients with moderate IPFD, with few to mild bleeding symptoms, may go undetected throughout their lives.

**Figure 1:**
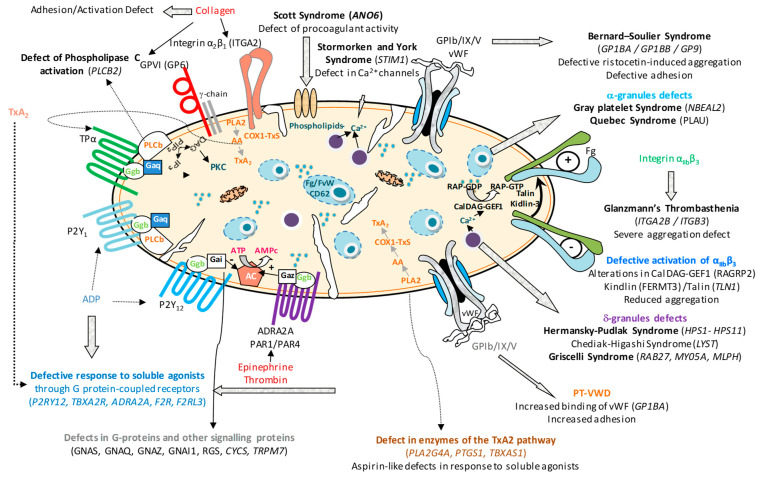
Overview of inherited platelet-function disorders (IPFDs) grouped by the affected protein or functional pathway, depicting genetic defects impairing platelet adhesion, activation, signalling and secretion.

Figure 1 shows the main IPFD and the affected platelet elements. Those inherited platelet disorders (IPD) that present platelet dysfunction, but are also associated with hereditary thrombocytopenia, such as, among others, Bernard–Soulier syndrome (BSS) (GP Ib/IX/V receptor defect) or gray platelet syndrome (alpha granule defect), are not detailed in this review.

## 2. Glanzmann’s Thrombasthenia

### 2.1. Introduction

Glanzmann´s thrombasthenia (GT) is a rare, autosomal recessive disease with an estimated worldwide incidence of 1/1,000,000, although it may be up to five times more prevalent in highly consanguinity populations [4]. It was first described in 1918 by the Swiss pediatrician Eduard Glanzmann syndrome in a patient with purpura and normal platelets in number and appearance. It is caused by genetic variants in the genes *ITGA2B* and *ITGB3*, both located on chromosome 17, which cause blockage of the synthesis of integrin subunits αIIb and β3, prevent the formation of the αIIbβ3 complex or interfere with its trafficking in megakaryocytes, or give rise to a dysfunctional αIIbβ3 [5,6,7]. This alteration makes it impossible or significantly difficult for fibrinogen and other integrin ligands to bind to platelets, resulting in a severe defect in platelet aggregation that significantly increases the risk of bleeding.

### 2.2. Clinical Manifestations

Although the clinical phenotype of GT is highly variable, it is usually characterized by a severe mucocutaneous bleeding diathesis from an early age, which occurs spontaneously or with minimal trauma. The most common bleedings are epistaxis (60–80%), gingivorrhagia (20–60%) and menorrhagia (60–90%) [1,4,8]. Epistaxis is very common, and can be severe, especially in children. Its frequency and intensity decrease with age. Heavy menstrual bleeding (HMB) is highly prevalent, affecting virtually all women with GT, and can cause severe bleeding, even from menarche [9]. Antifibrinolytic treatment, and oral contraceptives or intrauterine hormonal devices are frequently required for its control. There is a high risk of obstetric hemorrhage during labor and the puerperium [10]. It may occur in up to 75% of births, despite adequate hemorrhage control. Neuraxial anesthesia is usually contraindicated. Gastrointestinal bleeding may occur in 10–20% of cases, which is often difficult to manage. Intracranial hemorrhage may occur in 1–2% of patients. Hematuria and hemarthroses are uncommon. The psychosocial impact and impact on quality of life of frequent bleeding in these patients is important to note [8].

### 2.3. Diagnosis

In general, given the intense hemorrhagic symptoms that these patients present, their identification and diagnosis is usually made in the first years of life, although in 15% of cases it is delayed until the adolescence, and in rare cases it may not be diagnosed until adulthood [9,11].

**Figure 2:**
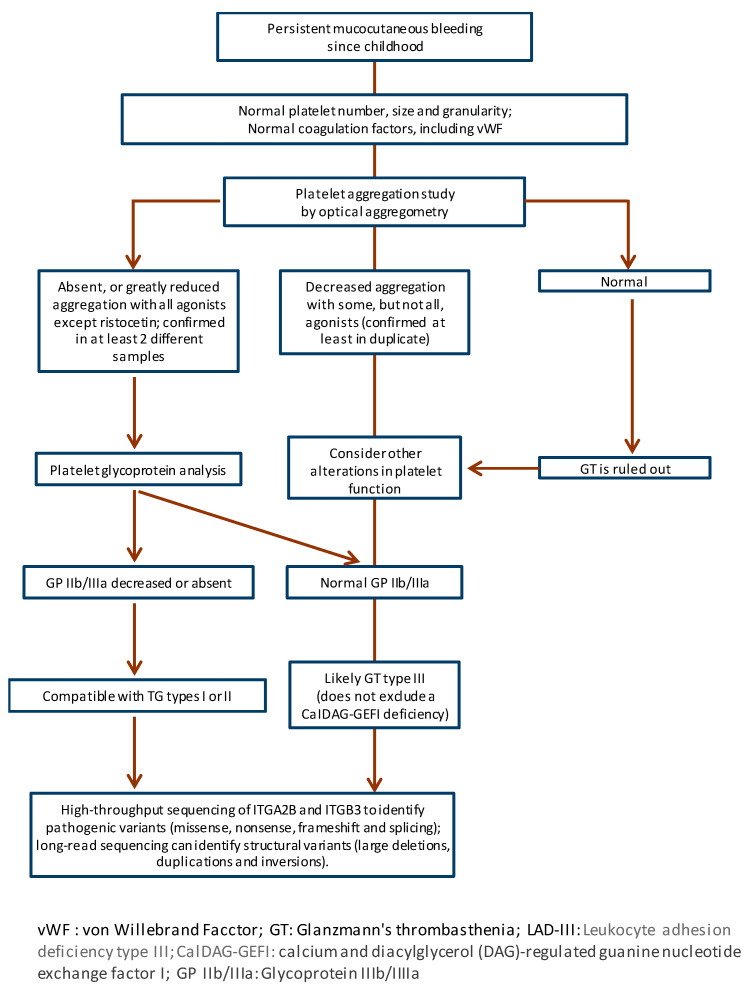
Diagnostic flowchart for Glanzmann’s thrombasthenia (GT) from clinical findings to genetic confirmation.

The diagnosis of GT should follow the steps recommended for the diagnosis of all IPD, starting with a detailed clinical evaluation of the patients [12]. Figure 2 shows a schematic flowchart of the diagnosis of this disease.

In the laboratory, a basic coagulation test is typically performed and shows no alterations, with both the platelet count and morphology being normal. If the PFA-100 system is used, very long obturation times (>300 s) are always observed with both cartridges (Collagen-ADP and Collagen-Epinephrine). A pathological result is also obtained if other point-of-care systems are used, such as T-TAS or Verify Now. Optical aggregometry in platelet-rich plasma (PRP) is the “gold standard” for the laboratory diagnosis of this disease. The classic pattern consists of no aggregation (<10%) to all platelet agonists (ADP, arachidonic acid, TRAP, collagen, and others), together with normal or moderately reduced aggregation/agglutination (up to ≈50%) to ristocetin. The reason is that ristocetin causes platelet agglutination not by fibrinogen binding to integrin αIIbβ3, but by VWF binding to the GP Ib/IX complex, whose expression is normal on GT platelets. Impedance aggregometry in whole blood, for example, with Multiplate equipment, is an alternative that also produces a pathological result in GT when using the soluble agonists mentioned above. The study of platelet glycoproteins expression by flow cytometry is probably the most widely used diagnostic method currently, due to its speed and because it requires a small volume of blood (≤100 μL). In this FC analysis, we can find in the GT three patterns of expression of GP IIb (CD41) and GP IIIa (CD61) (Table 1): (i) GT type I with absence of expression (<10%); (ii) GT type II with very reduced expression (10–25%); and GT type III or variant with moderate or even normal expression (>25%). The expression of other GPs (Ib/IX, GP VI, etc.) is normal in GT [1,2,4,9].

Glanzmann type I thrombastenia predominates globally (~75–80% of cases), followed by type II (~15%), with type III or variant being the rarest form (~5–10%). This general hierarchy can vary in certain population groups due to genetic factors (e.g., founder mutations) and endogamous practices [4,13,14].

The sequence analysis of the *ITGA2B* and *ITGB3* genes is necessary for the genetic diagnosis of GT, and is very useful for identifying carriers of causal mutations, reproductive counseling and/or for definitive prenatal or preimplantation diagnosis. As mentioned above, the inheritance of GT is typically autosomal recessive and symptomatic patients are homozygous for one pathogenic variant or, more rarely, double heterozygotes for two pathogenic variants each located on a single allele (in trans). Traditionally, sequencing of the *ITGA2B* and *ITGB3* genes was done by Sanger sequencing of DNA or cDNA, but it is currently addressed by high-throughput sequencing (HTS, also referred to as NGS), which is less laborious, faster, more accurate and even cheaper. In most patients, sequencing of these genes identifies the mutations responsible for the disease. They are usually nonsense, missense and insertions or deletions, or some changes affecting splicing sites. Large deletions, duplications, and complex rearrangements are rare [1,5,6,7,15]. Some complex structural variants are not easily detected by conventional sequencing based on short sequences, but resequencing of long sequences may facilitate their detection [16]. To date, up to 245 pathogenic or likely pathogenic variants in *ITGA2B* and 195 in *ITGB3* are reported in the ClinVar database (https://www.ncbi.nlm.nih.gov/clinvar/) (accessed 17 October 2025). In addition, another 669 variants of uncertain significance have been identified in these genes. Currently, expert groups such as those at ClinGen are making a great effort to define specific pathogenicity criteria for variants in these genes and to “cure” all known ones [17] (https://clinicalgenome.org/affiliation/50040/) (accessed 17 October 2025). 

Finally, it is worth mentioning that a pathology related to GT is autosomal dominant macrothrombocytopenia caused by heterozygous variants in the *ITGA2B* and *ITGB3* genes. Many of these mutations have a gain-of-function (GOF) character and cause a state of constitutive activation of the αIIbβ3 integrin, which however causes a platelet aggregation defect [18,19,20,21].

### 2.4. Treatment

The treatment of GT is similar to that of other severe IPDs. In addition to general hemostatic measures and the administration of antifibrinolytics, platelet transfusion is a mainstay therapy in the treatment of bleeding in GT. However, its use should be reserved for life-threatening bleeding, considering the high risk of developing alloantibodies against αIIb and β3, especially in patients with type I GT, and the possible refractoriness to subsequent transfusions [4,5,14,22,23,24,25]. If transfusion is needed, the use of HLA-matched, leukocyte-reduced platelets is recommended to minimize alloimmunization. Regular antibody screening is important in patients who have been transfused.

Administration of recombinant activated factor VII, can be used as an effective complement or alternative to platelet transfusion, in moderate or severe bleeding and perioperatively [26]. In our setting, it is indicated in patients with GT with previous or current rejection of platelet transfusions or where platelets are not readily available. It is administered intravenously at a dose of 90 µg (80–120 µg) per kg of body weight given at 2-h intervals (1.5–2.5 h). At least 3 doses should be administered to ensure effective hemostasis.

Hematopoietic stem cell transplantation may offer curative treatment for the disease, but due to its high morbidity and mortality, it should be restricted to severe cases [4,5,9,27,28]. Gene therapy using lentiviruses or CRISPR/Cas9-based technologies are very promising options, but, unlike other monogenic hemorrhagic diseases such as hemophilia, they are still in the preclinical research phase [9,29,30].

The most recent advance in the treatment of GT is sutacimig (formerly known as HMB-001), a bispecific antibody that both binds and stabilizes endogenous factor VIIa and binds to TLT-1, a protein present in activated platelets, helping to accelerate fibrin formation at sites of vascular injury and form a hemostatic plug [31]. This antibody is designed for subcutaneous administration, and the results of the Phase 2 trial show up to an 80% reduction in the rate of treated bleeding episodes in patients treated preventively with this antibody and an adequate safety profile. Sutacimig is thus positioned as the first effective prophylactic treatment that could transform the management of GT patients [31].

Finally, it should be mentioned that a pathology related to GT is autosomal dominant macrothrombocytopenia, caused by heterozygous variants in the *ITGA2B* and *ITGB3* genes. A significant proportion of these mutations are of the gain-of-function (GOF) type, resulting in constitutive activation of integrin αIIbβ3, which in turn leads to aggregation defects [18,19,20,21].

## 3. Platelet Function Disorders Due to Genetic Defects in Other Platelet Receptors

### 3.1. Deficiency of Glycoprotein VI (GP VI)

Glycoprotein VI (GP VI), encoded by the *GP6* gene on chromosome 19q13.4, is a key receptor in platelet adhesion and activation on collagen exposed after vascular injury. It is part of the immunoglobulin superfamily and is expressed exclusively in megakaryocytes and platelets. GP VI functions in conjunction with integrin α2β1, but is primarily responsible for intracellular signalling leading to platelet activation, granule secretion and thrombus formation. In addition, it interacts not only with collagen, but also with other adhesive proteins such as fibrin, which extends its role to thrombosis and inflammation [32].

Hereditary GP VI deficiencies are extremely rare and have been described mainly in the form of homozygous or compound heterozygous mutations in *GP6*, indicating that the common inheritance pattern is autosomal recessive. However, some heterozygous carriers may be symptomatic. Deletions, missense and nonsense mutations, and frameshift mutations that produce truncated or unstable proteins have been identified. Some patients show residual expression of GP VI but with defective function [33,34].

In terms of clinical characteristics, most patients present with mild to moderate mucocutaneous bleeding, including epistaxis, menorrhagia, or excessive post-surgical bleeding. No cases of severe intracranial haemorrhage have been reported. In laboratory tests, PFA-100 clotting times are usually prolonged with collagen/epinephrine cartridges. Platelet aggregation induced by collagen and its analogues is absent or markedly reduced, while responses to ADP, thrombin, or ristocetin are normal (Table 2). Flow cytometry shows reduced or absent GP VI on the platelet surface, and Western blot confirms total or partial loss of the protein. Underflow studies demonstrate defects in thrombus formation on collagen, with reduced adhesion and stability.

Data from patients and animal models confirm that the absence of GP VI protects against thrombosis without causing severe spontaneous haemorrhages, which has sparked interest in developing and evaluating anti-GP VI drugs as a new antithrombotic therapy, especially in ischaemic stroke [35].

### 3.2. Defects of the P2Y_12_ Receptor

The ADP P2Y_12_ receptor, coupled to Gi proteins, is essential for platelet aggregation mediated by this agonist, acting synergistically with the P2Y_1_ receptor. Upon activation, P2Y_12_ inhibits adenylate cyclase and potentiates PI3K-Rap1 signalling, ensuring complete platelet aggregation and the formation of a stable thrombus. Its functional relevance is well reflected in the therapeutic advantages provided by various anti-P2Y_12_ drugs such as clopidogrel, prasugrel, and ticagrelor. The *P2RY12* gene, located on chromosome 3q21-q25, encodes this P2Y_12_ receptor with 342 amino acids and transmembrane and extracellular domains [36].

Genetic deficiencies of the P2Y_12_ receptor (Table 2) are rare and heterogeneous, with a variable genotype-phenotype relationship. Their diagnosis requires platelet function studies, intracellular signalling tests, and genetic confirmation. Clinically, they present with spontaneous mucocutaneous haemorrhages that are generally moderate or severe, and excessive bleeding in response to haemostatic challenges such as trauma or surgery [1].

In the laboratory, platelets from patients with quantitative or functional P2Y_12_ deficiency show reversible or absent aggregation with ADP, even at high doses, while the response to thrombin, convulxin, or ristocetin is usually preserved or only partially affected (Table 2). A key diagnostic feature is the loss of adenylate cyclase inhibition downstream of the ADP-P2Y_12_-Gi axis, which can be assessed by the VASP protein phosphorylation test [1].

Molecularly, approximately a dozen pathogenic variants in *P2Y12* have been identified that are associated with quantitative and qualitative defects in the receptor. The former are usually due to deletions or frameshift mutations that produce truncated proteins or complete absence of the receptor (such as p.Gln98fs, p.Gly12fs, p.Thr126fs, p.Phe240fs), while qualitative defects result from missense mutations that allow receptor synthesis but alter its function (such as p.Arg256Gln, p.Arg265Trp, p.Pro258Thr, p.Lys174Glu, or p.Pro341Ala) [1,37,38].

Inheritance is mainly autosomal recessive, although heterozygous variants of apparent autosomal dominant inheritance have also been described, such as p.Arg265Pro, which give mild clinical phenotypes. These variants appear to exert a negative dominant effect, with overexpression and defective homodimer formation [37,38]. Curiously, it is not uncommon to find alterations in P2Y_12_ in patients with other bleeding disorders.

### 3.3. Deficiency of the TxA_2_ Receptor

The TPα receptor, encoded by the *TBXA2R* gene, is the main receptor for thromboxane A_2_ (TxA_2_), a key platelet agonist in haemostasis. Its activation, mainly coupled to Gq proteins, triggers Ca^2+^ mobilisation and platelet aggregation via the PLCβ pathway [39]. Hereditary TPα deficiency, first described in Japanese families in 1994, presents with mild to moderate mucocutaneous haemorrhages [1]. The laboratory phenotype is characterised by normal platelet count and morphology, but with a decreased aggregation response to arachidonic acid and TxA_2_ analogues (such as U46619 or I-BOP), and total or partial preservation for other agonists (Table 2).

At the genetic level, quantitative defects have been identified, such as the c.167dupG variant, which reduces receptor expression, and qualitative defects associated with missense mutations that alter signalling without affecting expression [40]. Among these, p.Arg60Leu stands out, located in the first intracellular loop of the receptor, with autosomal dominant inheritance and a dose-dependent effect: homozygotes with mild bleeding and heterozygotes with reduced response to TxA_2_. Other mutations such as p.Trp29Cys and p.Asn42Ser in transmembrane domain 1 (TMD1) can cause intracellular retention and dimerisation defects with a dominant negative effect, while p.Asp304Asn in TMD7 affects ligand binding and receptor activation. Taken together, these findings show that *TBXA2R* variants can affect the expression, dimerisation, ligand binding, or intracellular signalling of TPα, explaining the haemorrhagic phenotype and revealing regions critical for its function in platelet activation.

### 3.4. Alterations in the Ephrin Type B2 Receptor

EPHB2 is a receptor with tyrosine kinase activity whose role in platelets is not well understood, although it appears that it may regulate interactions between neighbouring cells. Interestingly, the alterations found to date do not affect this action of EPHB2, but they do affect its tyrosine kinase activity, which affects the phosphorylation of Lyn, Syk and FcR, the first steps in the GP VI collagen receptor signalling pathway [41,42,43].

Patients with deficiency of this receptor show reduced aggregation to ADP, collagen, arachidonic acid or TxA_2_ analogues and blockade of inside-out activation of integrin αIIbβ3 (Table 2). This is an autosomal recessive defect due to a nonsense mutation, with heterozygotes being asymptomatic.

The clinical picture is severe, with spontaneous and profuse bleeding after injury, although the case series is too small to clearly establish a phenotype [1,42].

## 4. Signal Transduction Defects

### 4.1. RASGRP2 (CalDAG-GEFI) Defect

In addition to intrinsic alterations in the genes encoding the two constituent proteins of integrin αIIbβ3, there are defects in other genes that can also alter integrin αIIbβ3 signalling [1,2,3]. This is the case of genetic variants in *RASGRP2* encoding the protein RASGRP2 or CalDAG-GEFI, the calcium- and diacylglycerol-regulated guanine nucleotide exchange factor [44,45]. RASGRP2 is abundantly expressed in platelets, and is essential in their rapid and calcium-dependent activation. It regulates the activation of Rap1, which in turn is involved in the initiation of integrin “inside-out” activation signaling, allowing binding to talin, which favors the switch to its active conformation. Typically, Rap1 activation occurs in two phases, a rapid but reversible phase mediated by RASGRP2 and increased cytoplasmic calcium, and a slower but sustained phase requiring PKC and the ADP receptor P2Y_12_. Functional deficiency of RASGRP2 results in reduced activation of integrin αIIbβ3, which impairs platelet aggregation and hemostatic clot formation. Mutations in RASGRP2 cause the so-called Bleeding disorder platelet type 18 (BDPLT18; OMIM 615888). This autosomal recessive disorder was first identified in humans by Canault M et al. in 2014, but approximately fifty unrelated families with this type of BDPLT18 have been described in the past decade [44,45,46,47].

Clinically, patients present with moderate/severe bleeding complications (epistaxis, hematomas, petechiae, exacerbated bleeding after minor trauma, etc.), which typically appear in children. Most affected women present with menorrhagia and significant postpartum hemorrhage, with a markedly negative impact on their quality of life. Chronic anemia resulting from persistent bleeding may require regular transfusions. Mild immunodeficiency may also sometimes be found, associated with deficiencies in the expression and/or activation of β1 and β2 integrins in neutrophils.

In the laboratory, patients with BDPLT18 show normal platelet count and morphology. PFA-100 assays show prolonged plugging times (>275–300 s) with collagen/ADP and collagen/epinephrine. Platelet aggregation assays show absent or markedly reduced aggregation with low doses of ADP, epinephrine, TRAP, or collagen, whereas the response with high doses or with PMA and ristocetin is normal (Table 2). Flow cytometry reveals normal glycoprotein expression, ruling out GT or BSS. Fibrinogen or PAC-1 binding and granule secretion are defective at low doses of agonists but normal at high doses or with PMA, consistent with defective RASGRP2-mediated Rap1 activation. Clot retraction is normal or only mildly impaired. Most patients have a quantitative defect in RASGRP2 demonstrated by Western blot in platelet lysates.

Genetic diagnosis of BDPLT18 is achieved by sequencing the RASGRP2 gene. To date, approximately thirty different genetic variants have been identified (missense, nonsense, splicing defects, frameshifts, deletions and insertions), some such as c.1280del are recurrent as they occur in different families. Most affect the CDC25 catalytic domain of RASGRP2, although there are variants in other regions, including three splicing variants. Most patients are homozygous, but there are also compound heterozygous carriers [44,45,46,47].

### 4.2. Defect or Leukocyte Adhesion Deficiency III (LADIII)

Kindlin-3, encoded by the *FERMT3* gene (chromosome 11q13.1), is one of three isoforms of kindlins, intracellular FERM domain-containing proteins that regulate integrin activation [48]. Kindlin-3 is exclusively expressed in hematopoietic cells, such as platelets and leukocytes, and is essential for the activation of integrin αIIbβ3. Upon agonist stimulation, talin binds to the proximal NPLY motif of integrin β3 and Kindlin-3 to the distal NxxY motif in the cytoplasmic tail, stabilizing their high-affinity conformation and promoting adhesion and signaling.

Pathogenic variants in *FERMT3* that alter expression or function of Kindlin-3, cause a severe autosomal recessive disease known as leukocyte adhesion deficiency type III (LADIII) (OMIM 612840) [49]. About 60 families with LADIII caused by about 35 different variants in *FERMT3* have been described, mostly nonsense and frameshift mutations (62%), and homozygous in 90% of cases, confirming autosomal recessive inheritance [50,51]. Clinically, it presents with hemorrhages similar to GT (epistaxis, petechiae, gastrointestinal hemorrhage, post-vaccination bleeding), frequent and severe infections (sepsis, pneumonia, osteomyelitis), leukocytosis, delayed healing and, occasionally, immune or skeletal alterations. In the laboratory, platelet aggregation with ADP, collagen, and thrombin is reduced, along with a normal response to ristocetin and normal glycoprotein expression, but activation defects (P-selectin). Neutrophils exhibit altered adhesion and migration (Table 2).

### 4.3. Defects in the TxA_2_ Pathway

Inherited defects in the thromboxane A_2_ (TxA_2_) pathway affect distinct stages of the synthesis or signaling of this eicosanoid essential for platelet activation [1,3,39,52]. The pathway begins with cPLA_2_α (*PLA2G4A*), a cytosolic phospholipase A_2_ that releases arachidonic acid from membrane phospholipids. Arachidonic acid is converted by cyclooxygenase-1 (COX-1) (*PTGS1*) to the cyclic endoperoxides PGG_2_/PGH_2_, which constitute the substrate for TxA_2_ synthetase (*TBXAS1*), responsible for generating TxA_2_, the effector molecule that acts on the TPα receptor (*TBXA2R*) already mentioned above.

Mutations in *PLA2G4A* (such as p.Ser111Pro and p.Arg485His) reduce or eliminate arachidonic acid production, with decreased platelet aggregation to collagen and ADP and mild to moderate mucocutaneous bleeding. Very few patients with pathogenic variants in *PTGS1* (*COX-1*), such as p.Trp322Ser or p.Asn143Ser, have been described, causing absent aggregation to arachidonic acid but a normal response to TxA_2_ analogues, reflecting a defect in synthesis but not in signaling [53,54]. TBXAS1 deficiency is associated with undetectable TxB_2_ production and lack of aggregation to multiple agonists; some variants are linked to Ghosal syndrome with osteosclerosis and variable bleeding diathesis [1,2,3].

Taken together, these defects show that any alteration of the cPLA_2_–COX-1–TBXAS1–TPα axis compromises the production or action of TxA_2_, with variable clinical repercussions but typically with mild or moderate mucocutaneous bleeding and characteristic functional findings in platelet aggregation and secretion studies.

### 4.4. Phospholipase C Defect

Phospholipase C (PLC) is a key enzyme in platelet activation, catalyzing the hydrolysis of phosphatidylinositol 4,5-bisphosphate (PIP2) into inositol-1,4,5-trisphosphate (IP3) and diacylglycerol (DAG). These second messengers regulate, respectively, the release of calcium from intracellular stores and the activation of PKC, essential steps for granule secretion, platelet shape change, and integrin activation required for aggregation. Platelets contain two subfamilies of PLC: PLCγ, which is mainly activated by ITAM receptors such as GP VI/FcRγ and integrins, generating more sustained calcium signals that are important for vascular integrity; and PLCβ, which is particularly activated by G protein-coupled receptors such as P2Y_12_ or TPα. PLCβ is responsible for rapid calcium peaks that are important for arterial thrombus formation [39]. Mucocutaneous bleeding and reduced platelet aggregation to multiple agonists have been described, associated with low PLCβ2 levels, defects in phosphatidylinositol metabolism, or variable [1,2,3,52]. Although mutations in genes such as *PLCB2* have been identified in some cases, we still generally do not know the genetics of patients suspected of suffering from this subtype of IPD.

### 4.5. Defect of G Proteins and Their Regulators

Heterotrimeric G proteins (Gα, Gβ, Gγ) are molecular switches that, upon activation of G protein-coupled receptors (GPCRs) such as P2Y_12_, TxA_2_ receptors, adrenaline, or thrombin receptors, promote the exchange of GDP for GTP at the Gα subunit, triggering multiple intracellular pathways. The four main families, Gαs, Gαi/o, Gαq/11, and Gα12/13, regulate cAMP synthesis, phospholipase C activation, increased intracellular calcium, and cytoskeletal reorganization. Several regulators of G proteins signalling (RGS) accelerate the hydrolysis of GTP to GDP at Gα, inactivating the signal, so their defects alter the duration and intensity of platelet activation.

Hereditary defects of G proteins and RGS constitute a heterogeneous group of IPFD due to alterations in intracellular signaling in platelets [1,2,3,52]. A few cases with variable clinical phenotypes have been described. Among them, one case with a 50% reduced Gαq expression and another with a moderate deficiency (75% expression) of Gαi1. Both showed a clinical phenotype with mild mucocutaneous bleeding, and defects in platelet aggregation and secretion, especially with weak agonists such as ADP and adrenaline. Sequencing of the corresponding genes did not identify candidate genetic variants, suggesting a defect at the level of transcription or mRNA stability [3]. In contrast, mutations in GNAS1 that increase Gαs activity (XLα polymorphism) cause hyperproduction of cAMP, decreased platelet adhesion, and excessive perioperative bleeding in some patients [55].

Regarding RGS regulators, a nonsense variant in RGS18 (p.Arg215 *) has recently been described, which causes loss of critical phosphorylation sites, maintains RGS18 in an active state (gain of function), and excessively inhibits G protein-coupled receptor signaling, with prolonged PFA-100 times, reduced secretion, and moderate mucocutaneous bleeding. Likewise, a variant in RGS2 (p.Gly23Asp) was associated with Gαs hypofunction and decreased cAMP in response to prostacyclins [52,56,57].

### 4.6. Other Platelet Signaling Defects

Apart from those mentioned above, there are other inherited disorders in proteins and signaling pathways critical for platelet function. These include alterations in Src family kinases (SFKs) that mediate signals downstream of αIIbβ3, GP VI-FcRγ and GP Ib-IX-V, the phosphatase CD148 which is a key regulator of SFKs, the cytoskeletal adapter FYB/ADAP, the GTPase CDC42 which also regulates cytoskeletal dynamics, or in PRKACG which encodes the catalytic γ subunit of protein kinase A. Patients with these abnormalities often exhibit defective platelet aggregation and/or secretion responses, as well as mild-to-moderate bleeding, depending on the affected protein and the type of mutation. Some patients also present with syndromic macrothrombocytopenia, which is characterised by cardiac, lymphatic, and neurological malformations [1,2,3,52]. Advances in high-throughput sequencing are enabling the identification of these rare variants, thereby expanding the clinical and molecular spectrum of IPFD associated with platelet signalling.

## 5. Defects of Platelet Granules

Another subgroup of IPFD is characterised by deficiencies of platelet granules, particularly dense (δ) granules. In this subgroup, platelet count and morphology are usually normal [1,2]. The main disorders in this subgroup are discussed below (Table 3).

### 5.1. Hermansky–Pudlak Syndrome

Hermansky–Pudlak syndrome (HPS) is a rare autosomal recessive disorder affecting the biogenesis and trafficking of lysosome-related organelles (LROs), such as melanosomes in melanocytes, dense granules (δ) in platelets, and Weibel-Palade bodies in the endothelium. LROs depend on vesicular transport from the Golgi apparatus and endosomes to acquire their specific contents. This process is regulated by the multiprotein complexes BLOC-1, BLOC-2, and BLOC-3 (Biogenesis of Lysosome-related Organelles Complex) and the AP-3 complex (Adaptor Protein complex 3) [58,59].

To date, 11 subtypes of HPS (HPS-1 to HPS-11) have been described, caused by mutations in the genes encoding the subunits of these multiprotein complexes that cause defects in the formation, transport or function of these organelles. Thus, in platelets there is an absence or reduction of δ granules that prevents the storage of ADP, ATP, serotonin and Ca^2+^, elements necessary for the amplification of platelet aggregation, favoring hemorrhagic diathesis. In melanocytes, the alteration of melanosomes explains albinism. Furthermore, in some subtypes of HPS, the affectation of LROs in epithelial cells, macrophages or lymphocytes contributes to granulomatous colitis and pulmonary fibrosis.

The global prevalence of HPS is low, estimated between 1:500,000 and 1:1,000,000. However, there are regions with genetic aggregation due to the founder effect, such as Puerto Rico, where HPS-1 (16 bp duplication) reaches a frequency of 1:1820 in the northwest and HPS-3 (3.9 kb deletion) reaches 1:4000 in the center of the island. Worldwide, the HPS Network (https://www.hpsnetwork.org/) (Accessed 17 October 2025). has registered around 1200 diagnosed patients, although underdiagnosis is suspected, especially for milder HPS subtypes or in areas with limited access to genetic diagnosis.

The most frequent clinical manifestations in HPS are oculocutaneous albinism with hypopigmentation, photophobia, nystagmus, foveal hypoplasia and decreased visual acuity, along with mild to moderate mucocutaneous hemorrhages such as epistaxis, ecchymosis, menorrhagia or postsurgical bleeding. A clear genotype-phenotype relationship is observed in the disease. Thus, in the subtypes HPS-1, HPS-4, HPS-2 and HPS-10, progressive pulmonary fibrosis may appear the main cause of morbidity and mortality, as well as granulomatous colitis and, in the case of HPS-2, immunodeficiency with recurrent infections and risk of hemophagocytic lymphohistiocytosis (HLH). In contrast, BLOC-2 (HPS-3, HPS-5, HPS-6) and BLOC-1 (HPS-7, HPS-8, HPS-9, HPS-11)-related subtypes typically present with milder phenotypes, limited to mucocutaneous bleeding and albinism.

Laboratory studies of HPS show a normal platelet count and clotting times. However, platelet aggregation reveals an absence or reduction of the second phase after stimulation with ADP or epinephrine, and a decreased response to collagen may also be present. Aggregation with ristocetin is normal. The definitive or gold standard diagnostic test is whole mount electron microscopy [12], demonstrating the absence or a significant reduction of δ granules. ADP/ATP content can also be assessed by lumiaggregometry, serotonin uptake and release can be assessed by the use of mepacrine to analyze secretion by cytometry, and CD63 or LAMP-1/-2 expression can be assessed by immunofluorescence of blood smears [60].

Genetic diagnosis, using gene panel or whole exome sequencing, is necessary to identify underlying genetic variants and identify the HPS subtype, something of crucial prognostic importance considering the genotype-clinical phenotype relationship mentioned above. Nearly 300 distinct mutations have been described in the 11 genes, including nonsense, frameshift, splicing, and missense variants. As mentioned above, founder mutations in HPS1 and HPS3 explain the high prevalence in Puerto Rico. In cases with strong clinical suspicion but negative genetic diagnosis with standard short read sequencing analysis, long-read sequencing can detect previously unidentified molecular alterations [16].

Treatment is primarily supportive. Hemorrhagic manifestations are managed with desmopressin (DDAVP), antifibrinolytics such as tranexamic acid, and, in severe or surgical cases, platelet concentrates. For pulmonary fibrosis, antifibrotics such as pirfenidone are being studied, and lung transplantation is an option in advanced disease. In HPS-2, immunodeficiency requires antimicrobial prophylaxis and immunological monitoring. Advanced therapies, such as gene therapy and lysosomal trafficking modulators, are being explored, although still in experimental phases [61].

### 5.2. Chediak–Higashi Syndrome

Chediak–Higashi syndrome (CHS) is another very rare autosomal recessive disorder (<500 cases described), with a similar etiopathogenesis to HPS [62,63]. Thus, the disease is caused by biallelic mutations in the *LYST* gene (1q42.3), which encodes the LYST protein (Lysosomal Trafficking Regulator) of the BEACH (Beige and Chediak–Higashi) family, involved in the biogenesis and trafficking of LROs in immune cells (NK cells, cytotoxic T lymphocytes), melanocytes, platelets and neurons. Dysfunction of LYST results in giant intracytoplasmic granules and defective lysosomal exocytosis of perforin and granzymes, leading to immunodeficiency. It also causes pigmentation abnormalities due to abnormal melanosome distribution, platelet dysfunction that predisposes to bleeding, and neuronal lysosomal accumulation with impaired autophagy contributing to progressive neurodegeneration. Clinically, CHS presents a heterogeneous spectrum and two modalities have been commonly described. A classic form, associated with nonsense or frameshift mutations, which usually debuts in childhood with partial oculocutaneous albinism, severe immunodeficiency with pyogenic infections, hemorrhagic diathesis and high risk (>80%) of HLH, which is the main cause of death before adolescence. The atypical form of CHS, associated with missense or in-frame mutations, usually manifests later, with less severe infections but with progressive neurodegeneration (peripheral neuropathy, cerebellar ataxia, parkinsonism, cognitive impairment, spastic paraplegia). This typical/atypical disease classification is less rigid today, since both phenotypes can overlap.

The laboratory diagnosis of CHS is based on the identification of giant granules in neutrophils, lymphocytes, or hair cells using blood smears or electron microscopy. Platelet defects are very similar to those of HPS, including the marked reduction in δ granules demonstrable by whole mount electron microscopy, immunofluorescence smear or cytometry test (CD63, mepacrine), or the absence of the second wave of aggregation after ADP or epinephrine [12].

Genetic confirmation is achieved by identifying recessive variants in *LYST*. Nearly 200 have been described, most commonly frameshift (41%) and nonsense (30%) variants, but also missense, splicing or small deletions, in-frame and loss-of-homing variants [64] residual protein functionality give rise to CHS with milder phenotypes although commonly associated with neurodegeneration.

The treatment of choice is allogeneic hematopoietic stem cell transplantation (HSCT), which corrects immunodeficiency and, if performed early, prevents HLH, but does not halt neurodegeneration. Symptomatic management includes immunosuppressive therapy for HLH (e.g., steroids and etoposide), antibiotics for infections, and neurological and hematological support. There are currently no specific therapies for neurodegeneration, although gene editing strategies and lysosomal modulators are being explored in preclinical models [62,63].

A very rare disease related with defects in secretion of α and δ granules and lysosomes, and with clinical similarities, is familial hemophagocytic lymphohistiocytosis. However, in this case the cause is recessive mutations in the genes *UNC13D*, *STX11* and *STYXBP2* [1].

### 5.3. Griscelli Syndrome

Griscelli syndrome (GS) is another rare autosomal recessive disorder characterized by cutaneous hypopigmentation and silver hair, associated with neurological and immunological alterations of variable severity [2,65]. Three subtypes are distinguished according to the mutated gene: GS type 1, caused by mutations in MYO5A (myosin Va, an actin motor protein involved in the intracellular transport of organelles), presents with severe neurological disorders with a usually unfavorable prognosis, such as psychomotor retardation, seizures and ataxia, but without immunodeficiency or risk of hemophagocytosis; GS type 2, due to mutations in RAB27A (a GTPase Rab essential in lysosome and cytotoxic granule exocytosis), is associated with severe immunodeficiency, NK and cytotoxic T cell dysfunction, and a high risk of HLH, with secondary neurological symptoms; and type 3 GS, with mutations in MLPH (melanophilin, an adapter protein that binds myosin Va and RAB27A in melanosome transport), is characterized by isolated pigmentary alteration without neurological or immune involvement. Diagnosis combines hair shaft microscopy, which reveals irregular clumps of melanin, with genetic confirmation. The treatment of choice for type 2 GS is early hematopoietic stem cell transplantation, accompanied by antimicrobial prophylaxis and HLH therapy, while types 1 and 3 have no specific therapeutic options.

### 5.4. Arthrogryposis Syndrome, Renal Dysfunction and Cholestasis (ARC Syndrome)

It is an autosomal recessive disease caused by pathogenic genetic variants (about 150 in ClinVar; https://www.ncbi.nlm.nih.gov/clinvar/) (Accessed 17 October 2025) in the *VPS33B* and *VIPAS39* genes, which encode the VPS33B (Sec1/Munc18 family) and VPS16B proteins, respectively. These proteins form a vesicular trafficking complex essential for the biogenesis of α-granules in megakaryocytes/platelets and other epithelial organelles, which explains the characteristic clinical triad—arthrogryposis congenita, renal tubulopathy, and cholestasis with low GGT—together with the bleeding diathesis [59,66].

The platelet count is usually normal, and moderate thrombocytopenia, although present, is not a defining feature. Platelet dysfunction and associated bleeding diathesis in this syndrome are attributed to abnormal α-granule biosynthesis or exocytosis. Episodes ranging from postoperative bleeding to life-threatening spontaneous hemorrhage have been described. The presence of agranular platelets on the smear and the absence of α-granules on electron microscopy are key findings for diagnostic guidance. Platelets also show a marked deficiency of α-granule markers, such as P-selectin, on immunofluorescence staining of blood smears or flow cytometry Platelet aggregation may be reduced with agonists such as ADP, epinephrine, or collagen, although it is almost normal in some patients.

### 5.5. Non Syndromic Granule Deficiencies

Unlike the above, non-syndromic platelet storage pool disorders (δ-SPD) are a frequent cause of bleeding diathesis, with estimates between 10–30% (depending on the series) of all congenital platelet defects, although their exact prevalence is uncertain and many patients may go unnoticed [1,2]. The intensity of bleeding is usually mild or moderate (epistaxis, ecchymosis, menorrhagia), although in cases of surgery or trauma it can be severe.

Platelet count and basic coagulation are normal, so diagnosis requires specialized techniques such as electron microscopy, which shows reduced or absent dense granules, and platelet function tests (aggregometry, mepacrine, serotonin release, cytometry for CD63). The molecular basis of δ-SPD remains largely unknown, and most cases are considered idiopathic. Some have been linked to alterations in the MRP4 transporter, involved in the incorporation of nucleotides into δ-granules. Others combine deficiency of both δ and α granules and have recently been linked to alterations in the GFI1B transcription factor gene.

In this category, we can also mention Quebec Syndrome (QS, 601709), a platelet hemorrhagic disorder with impairment of granule contents. This rare disorder is caused by a heterozygous tandem duplication of the *PLAU* gene (#191840, chromosome 10q22), which behaves as a gain-of-function mutation, increasing intraplatelet levels of PLAU and plasmin that degrades α granule proteins. Affected individuals, almost exclusively in Canada, do not have systemic fibrinolysis, but do have a predisposition to delayed-onset bleeding after a hemorrhage challenge, such as surgery [67,68].

## 6. Defects in Membrane Phospholipids and/or Procoagulant Activity: Scott Syndrome

Scott syndrome (SS) is an extremely rare thrombocytopathy characterized by an isolated deficiency of platelet procoagulant activity due to the absence of phosphatidylserine (PS) exposure on the membrane upon activation [1,69]. PS provides an essential anionic surface for the assembly of tenase and prothrombinase complexes, increasing thrombin generation thousands of times. Under normal conditions, PS is confined to the inner face of the membrane by flippase activity and, upon strong stimulation with increased Ca^2+^, the scramblase TMEM16F (encoded by *ANO6*) redistributes PS to the outer face. In Scott syndrome, this scramblase is inactive, resulting in the abolition of PS exposure and the release of procoagulant microparticles.

Clinically, patients with SS present with mild to moderate bleeding, often following surgery or childbirth, with mucosal haemorrhages and menorrhagia in some women, but without severe spontaneous bleeding. To date, only a few families (7 patients) have been described worldwide [70,71], suggesting that it is underdiagnosed due to the mild clinical presentation and the lack of routine and/or specific diagnostic tests.

In the laboratory, platelet counts, adhesion, aggregation, and secretion are generally normal, but PS exposure, as measured by annexin V flow cytometry after stimulation with collagen/thrombin or calcium ionophore, is markedly reduced or absent. Decreased thrombin generation in plasma and whole blood, as well as impaired release of PS-containing extracellular vesicles, is also observed.

Genetically, eight distinct mutations have been identified in the *ANO6* gene (TMEM16F), all of which are autosomal recessive and associated with loss of function of phosphatidylserine scramblase. They include frameshift mutations by short deletions or insertions that cause protein truncation, nonsense with premature stop codons, splicing disorders, and, to a lesser extent, in-frame deletions with residual expression of the protein. All produce an absence or marked reduction in phosphatidylserine exposure after platelet activation, resulting in a mild to moderate hemorrhagic phenotype with normal coagulation and aggregation tests but defective procoagulant activity.

Regarding treatment, there is no specific therapy; management is symptomatic, with prophylactic platelet transfusions in high-risk bleeding situations, such as surgery or childbirth. Gene therapy studies have been proposed, but this is still in preclinical research worldwide [70,71].

## 7. General Approach to IPFD Treatment

In the above description of some of the IPFD, we have already discussed specific aspects of their treatment. As a general approach, we can say that the treatment of IPFD should be adapted to the severity of the bleeding, the type of defect, and the specific clinical situations. Personalized medicine is therefore especially recommended [1,2,9,52,61,72,73,74,75].

Most patients present with mild to moderate mucocutaneous bleeding, effectively controlled with local measures, antifibrinolytics such as tranexamic acid and, in some cases, desmopressin (DDAVP), especially useful in minor procedures or minor bleeding, although it is not effective in all platelet defects. For major surgeries or severe bleeding, platelet transfusion is the treatment of choice, preferably administering HLA-compatible platelets to reduce the risk of alloimmunization. In patients with anti-platelet antibodies or refractoriness, as many patients with Glazmann’s thrombasthenia, recombinant activated factor VII has proven useful as a rescue therapy.

Allogeneic hematopoietic stem cell transplantation is currently the only curative option for some multisystem, life-threatening IPFD, especially those with severe hematological and immunological involvement such as LAD-III or CHS. Gene therapy is under investigation, with promising preliminary results in experimental models, but has not yet reached broad clinical application.

The most innovative therapies employ bispecific antibodies that facilitate coagulation on the platelet surface and stable thrombus formation in areas of vascular injury [31].

Current management also includes individualized prophylaxis for invasive procedures, pregnancy, and childbirth, with multidisciplinary planning involving hematologists, anesthesiologists, and obstetricians. Early genetic diagnosis aids in the design of more tailored prevention strategies and appropriate family counseling.

## Figures and Tables

**Table 1:** Types of Glanzmann’s thrombasthenia.

	Level of αIIbβ3	Frequency Relative	Platelet Phenotype
Type I	<5%	75–80%	Absence of platelet aggregation and clot retraction. Empty platelet fibrinogen storage pool
Type II	5–25%	15%	Absence of platelet aggregation. Partial or normal clot retraction Slightly reduced platelet fibrinogen storage pool
Type III	25–100% (qualitative defects)	5–10%	Absence of platelet aggregation. Clot retraction and platelet fibrinogen storage pool are highly variable

**Table 2:** Main deficits of soluble agonist receptors, signal transmission and procoagulant activity.

Pathology (OMIM)	Genes	Inheritance	Hemorrhagic Phenotype	Platelet Phenotype
P2Y_12_ receptor (OMIM # 609821)	*P2RY12*	AD/AR	Mild-moderate	Inhibited aggregation to ADP. Reduced VASP phosphorylation.
TxA_2_ receptor (OMIM # 614009)	*TBXA2R*	AD/AR	Mild-moderate	Reduced aggregation to AA and TxA_2_ analogues
Ephrin receptor type B2 (OMIM # 618462)	*EPHB2*	AR	Moderate-severe	Aggregation and granule release inhibited with various agonists
GP VI receptor (OMIM # 614201)	*GP6*	AR	Mild-moderate	Absence of aggregation to collagen and GP VI agonists
CalDAG-GEFI (OMIM # 615888)	*RASGRP2*	AR	Moderate-severe	Reduced aggregation with low concentrations of collagen, arachidonic acid or ADP. Decreased activation of α_IIβ_β_3_
Leukocyte adhesion defect type III (kindlin-3) (OMIM # 612840)	*FERMT3*	AR	Severe	Similar to Glanzmann’s thrombasthenia
Scott syndrome (OMIM # 262890)	*AN06*	AR	Moderate-severe	Impaired annexin V binding and microparticle release

AA: Arachidonic acid; AD: Autosomal Dominant; AR: Autosomal recessive; GP VI: glycoprotein VI; TxA_2_: thromboxane A2; VASP: Vasodilator-stimulated phosphoprotein.

**Table 3:** Platelet granule deficiencies.

Pathology (OMIM)	Genes Involved	Inheritance	Hemorrhagic Phenotype	Platelet Phenotype
Hermansky–Pudlak syndrome HPS1: (OMIM # 203300) HPS2: (OMIM # 608233) HPS3: (OMIM # 614072) HPS4: (OMIM # 614073) HPS5: (OMIM # 614074) HPS6: (OMIM # 614075) HPS7: (OMIM # 614076) HPS8: (OMIM # 614077) HPS9: (OMIM # 614171) HPS10: (OMIM # 617050) HPS11: (OMIM # 619172)	*HPS1* *HPS2: AP3B1* *HPS3* *HPS4* *HPS5*, *HPS6* *HPS7: Dysbindin* *HPS8: BLOS3* *HPS9: Pallidine* *HPS10: AP3D1* *HPS11:BLOC1S5*	AR	Mild-moderate	Reduction or absence of δ granules confirmed by different techniques
Chediak–Higashi syndrome CHS: (OMIM # 214500)	*LYST*	AR	Mild-moderate	Reduction or absence of δ granules confirmed by different techniques
Griscelli syndromes (SG1: OMIM # 214450) (SG2: OMIM # 607624) (SG3: OMIM # 609227)	*RAB27* *MY05A* *MLPH*	AR	Mild	Reduction or absence of δ granules confirmed by different techniques
Familial hemophagocytic lymphohistiocytosis (OMIM # 267700)	*UNC13D* *STX11* *STYXBP2*	AR	Mild	Deficiency of δ and α granules, and lysosomes
Combined δ and α granule defects		AR/AD	Mild-moderate	Deficit of content or release of granules

AD: Autosomal Dominant; AR: Autosomal recessive; HPS: Hermansky–Pudlak syndrome; δ and α granules: dense and alpha platelet granules.

## Data Availability

No new data were created or analyzed in this study.

## Abbreviations

AA	arachidonic acid
AD	autosomal dominant
ADP	adenosine diphosphate
AR	autosomal recessive
ATP	adenosine triphosphate
BDPLT18	bleeding disorder platelet type 18
BLOC	biogenesis of lysosome-related organelles complex
BSS	Bernard–Soulier syndrome
CDC42	cell division control protein 42 homolog
CHS	Chediak–Higashi syndrome
COX-1	cyclooxygenase-1
DAG	diacylglycerol
DDAVP	desmopressin
DNA	deoxyribonucleic acid
EPHB2	ephrin type B2 receptor
FcR	Fc receptor
FC	flow cytometry
FERM	four-point-one: ezrin: radixin: moesin domain
FYB/ADAP	FYN-binding protein/adhesion and degranulation-promoting adapter protein
GDP	guanosine diphosphate
GEF	guanine nucleotide exchange factor
GGT	gamma-glutamyl transferase
Gi	inhibitory G protein alpha subunit
GP	glycoprotein
GPCR	G protein-coupled receptor
GPIIb/IIIa	integrin αIIbβ3
GPIb/IX/V	glycoprotein Ib-IX-V complex
Gq	G protein alpha q subunit
GT	Glanzmann thrombasthenia
GTP	guanosine triphosphate
HMB	heavy menstrual bleeding
HLH	hemophagocytic lymphohistiocytosis
HPS	Hermansky–Pudlak syndrome
HSCT	hematopoietic stem cell transplantation
HTS	high-throughput sequencing
ITGA2B	integrin subunit alpha IIb
ITGB3	integrin subunit beta 3
LAD-III	leukocyte adhesion deficiency type III
LRO	lysosome-related organelle
LYST	lysosomal trafficking regulator
mRNA	messenger ribonucleic acid
NGS	next-generation sequencing
OMIM	Online Mendelian Inheritance in Man
PCR	polymerase chain reaction
PFA-100	platelet function analyzer-100
PIP2	phosphatidylinositol 4:5-bisphosphate
PKC	protein kinase C
PLC	phospholipase C
PMA	phorbol myristate acetate
PS	phosphatidylserine
PTGS1	prostaglandin-endoperoxide synthase 1
QS	Quebec syndrome
RASGRP2	RAS guanyl-releasing protein 2
RGS	regulator of G-protein signaling
RNA	ribonucleic acid
SFK	Src family kinases
SS	Scott syndrome
TBXA2R	thromboxane A2 receptor
TMEM16F	transmembrane protein 16F (Anoctamin 6)
TPα	thromboxane receptor alpha isoform
TRAP	thrombin receptor-activating peptide
TxA2	thromboxane A2
VASP	vasodilator-stimulated phosphoprotein
VWD	von Willebrand disease
VWF	von Willebrand factor
